# 3-dimensional digital reconstruction of the murine coronary system for the evaluation of chronic allograft vasculopathy

**DOI:** 10.1186/s13000-015-0248-6

**Published:** 2015-03-28

**Authors:** László Fónyad, Kazunobu Shinoda, Evan A Farkash, Martin Groher, Divya P Sebastian, A Marcell Szász, Robert B Colvin, Yukako Yagi

**Affiliations:** 1st Department of Pathology and Experimental Cancer Research, Semmelweis University, Budapest, Hungary; Department of Pathology, Massachusetts General Hospital, Harvard Medical School, Boston, MA USA; microDimensions GmbH, Munich, Germany; 2nd Department of Pathology, Semmelweis University, Budapest, Hungary

**Keywords:** Digital slides, 3D, CAV, Serial sectioning, Neointimal volume index

## Abstract

**Background:**

Chronic allograft vasculopathy (CAV) is a major mechanism of graft failure of transplanted organs in humans. Morphometric analysis of coronary arteries enables the quantitation of CAV in mouse models of heart transplantation. However, conventional histological procedures using single 2-dimensional sections limit the accuracy of CAV quantification. The aim of this study is to improve the accuracy of CAV quantification by reconstructing the murine coronary system in 3-dimensions (3D) and using virtual reconstruction and volumetric analysis to precisely assess neointimal thickness.

**Methods:**

Mouse tissue samples, native heart and transplanted hearts with chronic allograft vasculopathy, were collected and analyzed. Paraffin embedded samples were serially sectioned, stained and digitized using whole slide digital imaging techniques under normal and ultraviolet lighting. Sophisticated software tools were used to generate and manipulate 3D reconstructions of the major coronary arteries and branches.

**Results:**

The 3D reconstruction provides not only accurate measurements but also exact volumetric data of vascular lesions. This virtual coronary arteriography demonstrates that the vasculopathy lesions in this model are localized to the proximal coronary segments. In addition, virtual rotation and volumetric analysis enabled more precise measurements of CAV than single, randomly oriented histologic sections, and offer an improved readout for this important experimental model.

**Conclusions:**

We believe 3D reconstruction of 2D histological slides will provide new insights into pathological mechanisms in which structural abnormalities play a role in the development of a disease. The techniques we describe are applicable to the analysis of arteries, veins, bronchioles and similar sized structures in a variety of tissue types and disease model systems.

**Virtual slides:**

The virtual slide(s) for this article can be found here: http://www.diagnosticpathology.diagnomx.eu/vs/3772457541477230.

## Background

Although great strides have been made in preventing acute transplant rejection, many transplants fail at late time points due to chronic rejection. Chronic allograft vasculopathy (CAV) is a type of rejection resulting in neointimal thickening, the progressive narrowing of vascular lumens secondary to inflammation and fibrosis, is a common cause of graft dysfunction in hearts and other organs. The pathogenesis of chronic graft rejection is an active area of basic and translational study with the potential to improve transplantation outcomes. Mouse models of cardiac allotransplant frequently rely on the evaluation of morphologic changes in coronary arteries as a surrogate endpoint in determining graft rejection, or as a means of exploring the mechanisms of CAV [1-7]. The neointimal thickness of coronary arteries as measured by conventional light microscopy is used as a quantitative measurement of the severity of CAV [8,9]. However there are many limitations and pitfalls with this method.

One practical problem is that mouse coronary arteries are small, about 0.1 mm in diameter, similar in size to intramyocardial vessels in the human and about the width of a human hair [10]. In addition, the preferential and earliest sites of transplant arteriopathy in mouse heart allografts are proximal segments beginning with the coronary orifices a region 1–2 mm in length. Detecting this site on histological sections for morphometric analysis is very challenging, even for skilled histotechnologists.

**Figure 1:**
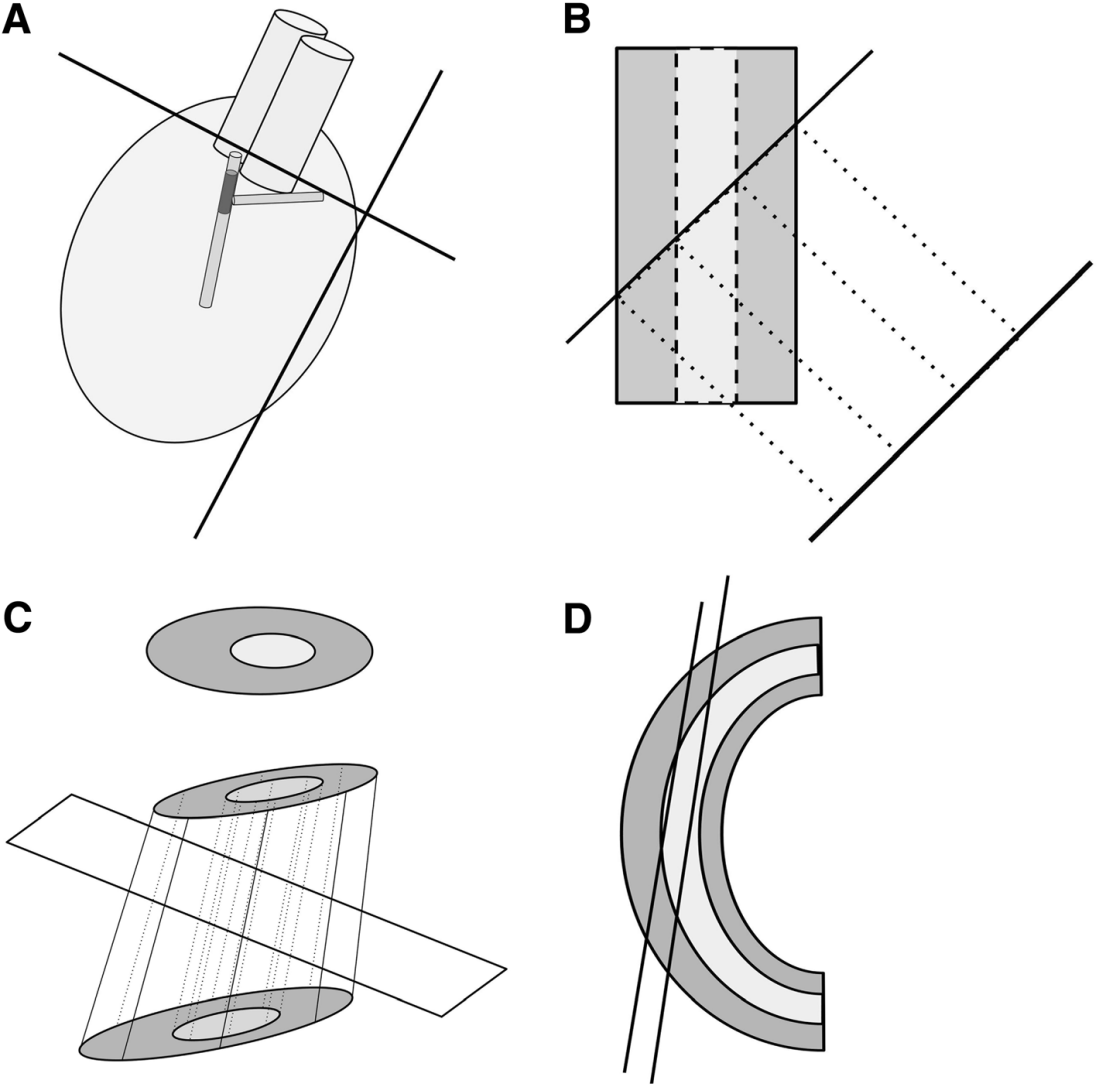
**Schematic representation of a mouse heart, area of vasculopathy and explanation of the neointimal index and its limitations. A**. Model of mouse heart with coronaries targeted for histologic analysis in dark grey. Because of their small size and variation in origin and path, there is a real possibility of failing to capture the area in histologic sections (lines) or missing true cross sections. **B**. An oblique cut through a vessel increases the apparent neointimal thickness in the linear dimension. **C**. For straight vessels, if the internal elastic lamina and residual lumens are approximately elliptical, the calculated neointimal index should give comparable results independent of the cutting angle of the vessels. **D**. For curved vessels, tangential sectioning close to the vessel wall will cause a falsely elevated neointimal index.

The orientation of the embedded tissue plays an important role in successfully sampling CAV. Since the area of interest is small and practically impossible to identify with the naked eye during embedding, it is challenging to orient samples to obtain exact coronal sections of the coronary arteries (Figure 1A). For improperly oriented specimens, the coronary arteries could be cut through in as few as twenty 5 μm sections. Improper orientation during embedding and sectioning raises the likelihood of tangential sectioning of the coronary arteries and subsequently mismeasuring the neointimal thickness (Figure 1 B-D).

Coronary artery sections obtained from oblique cuttings display inaccuracy of measurements that lead to overestimation or underestimation of the neointimal lesions. By simple Euclidean geometry, tangential sections increase the absolute width of the maximal neointimal thickening (Figure 1B). To address the pitfall when measuring the neointima in single dimension, Armstrong et al. developed the neointimal index (NI), which is defined as neointimal area divided by neointimal area plus luminal area multiplied by 100 [2]. (Figure 1C). Although this method yields an accurate result for straight vessels when cut either in true cross-section or at an oblique angle, the authors in their original analysis intentionally included as a criterion vessels that were not cut obliquely.

However, the neointimal index obtained from a single histologic cross section is unable to accurately address the problem of measuring the neointima formation in an obliquely cut curved vessel. Figure 1D. demonstrates how tangential cuts through curved vessels can have widely varying NI ratios, even if the ratios of the lengths or the areas are calculated.

In this paper we describe a sophisticated tool of digital pathology that ensures sectioning of minute structures such as the mouse coronary system and the 3D reconstruction of digital slides of serial sections that accurately quantitates CAV lesions in mouse heart allografts.

## Methods

### Mice

C57BL/6 (H-2b), B10.BR (H-2 K), B6.129S7 (Rag1tm1Mom(B6.RAG1−/−, H-2b) and C.FVB-Tg Itgax-DTR/EGFP 57Lan/J (C.CD11c DTR, H-2d) mice were purchased from The Jackson Laboratory (Bar Harbor, ME). The mice were included in the experiments between the ages of 8 to 12 weeks. All mice were maintained under pathogen-free conditions in filter-top cages throughout the experiments with an automatic water system and were cared for according to methods approved by the American Association for the Accreditation of Laboratory Animal Care.

### Polyclonal antibodies

For the production of polyclonal antibodies to B10.BR, B6 mice received a skin graft from B10.BR mice. After skin grafts were completely rejected, B6 mice were then boosted with 1 to 2 106 B10.BR splenocytes every two weeks. Sera were collected from the third time of booster immunization and used as polyclonal antibodies to B10.BR. The specificity of these antibodies was confirmed by analyzing thymocytes from B6, B10.BR and DBA/2 (H-2d) on a flow cytometer (data not shown).

### Heterotopic heart transplantation

Transplantation of mice heart grafts was performed according to the previously described microsurgical technique [11]. In brief, the aorta and pulmonary artery of heart grafts were anastomosed to the recipient infrarenal aorta and inferior vena cava, respectively, in an end-to-side fashion. Cold ischemic times of less than 25 min were maintained throughout. In order to develop lesions of CAV, two distinct models were conducted. The recipient in the first model was B6.RAG1−/− lacking mature T cells or B cells, which was transplanted a heart allograft from B10.BR and treated with sera containing polyclonal antibodies to B10.BR in 0.2 ml in each time, beginning the day after transplantation and continuing three times a week until completion of the experiment. This model was applied to develop CAV via antibody and NK cells. Several studies showed that donor specific monoclonal antibodies to class I MHC antigen promote CAV lesions in transplanted heart allografts in coordination with NK cells [6,7]. The second model was the combination between a male C.CD11c DTR to a female C.CD11c DTR, which was exploited to focus on the difference in H-Y minor histocompatibility antigen. The male-specific HY minor histocompatibility was first detected by Eichwald and Silmser [12] and the development of CAV lesions in a mouse heart transplant model incompatible for HY antigen was reported previously [13].

### Sample preparation

The heart grafts were removed on day 28, fixed in 10% formalin. Three different types of samples were prepared; the allografts from the first and second models described above and native heart from a B10.BR male mouse and were named as Sample A, B and C, respectively. Sample A, B was embedded in high melting point paraffin (58°C, Tissue-Prep, Fisher Scientific, Idaho, USA) required for automated sectioning. For sectioning the Kurabo AS-200S Automated Tissue Sectioning System (Kurabo Industries, Osaka, Japan) was used. The samples were sectioned using the default parameter settings of the device. Sample C was embedded in regular paraffin and sectioned manually. The thickness of the sections was 4 μm each. The sections were hematoxylin-eosin (H&E)-stained manually in batches.

### Slide digitization and 3D reconstruction

Glass slides were scanned by two different methods. First, bright field scanning was performed with Pannoramic Scan (3DHISTECH, Budapest, Hungary) using Plan-Apochromat 20x magnification objective, a 0.63x camera adapter magnification and 1x Optovar magnification with a Hitachi HV F22CL camera, resulting in 0.369 μm/pixel resolution. To enhance image quality, extended focus mode was used with 5 additional focus depths, each of 0.2 μm thickness value above and below the calculated focus levels. Image compression was JPEG; the degree of compression was minimal and set to 90 JPEG Quality Factor. Second, fluorescent scanning was performed on selected slides after locating regions of interest. For fluorescence scanning a Pannoramic Scan device with HXP 120 illuminator was used. The digital slides were in .mrxs format.

The μCore software was used to generate 3D reconstructions (MicroDimensions, Germany, μCore was a working title of the software, currently is called Voloom). This software accepts digital slides in .mrxs format and has algorithms for automatic stacking and alignment. Whole digital slides were used for creating an overview of the serial sections. Using the overview stacks, the sections containing vascular structures were identified, beginning with the coronary artery orifices and extending to major bifurcations, and these were used to define areas of interests. Limiting alignment and 3D image calculations to only the region of each slide that contained the coronaries resulted in a pronounced improvement in the speed of reconstruction, enabling much higher magnification and finer resolution. The μCore software also allows performance of virtual rotation and re-sectioning of a reconstructed sample, thus generating virtual, true cross sections of the coronary arteries. NI was calculated on these virtual sections using ImageJ software. The μCore software also permits segmentation of objects in 3-dimensions and calculation of volumes in cubic micrometers. Measuring the CAV as ratio of volumes of a given coronary portion minimizes the limitations of individual section orientation as described in the Introduction.

### Statistical analysis

Statistical analysis was performed using IBM SPSS Statistics 20 Software Package (IBM, Inc., Armonk, NY, USA). For the correlation of the continuous variables paired-samples *t*-test was utilized /t(df)/. Independent samples *t*-test and general linear model multivariate test was used to assess difference in distribution between the groups of the continuous value groups. P-value of less than 0.05 was considered significant.

## Results and discussion

### Virtual cross sectioning of murine coronary arteries

**Figure 2:**
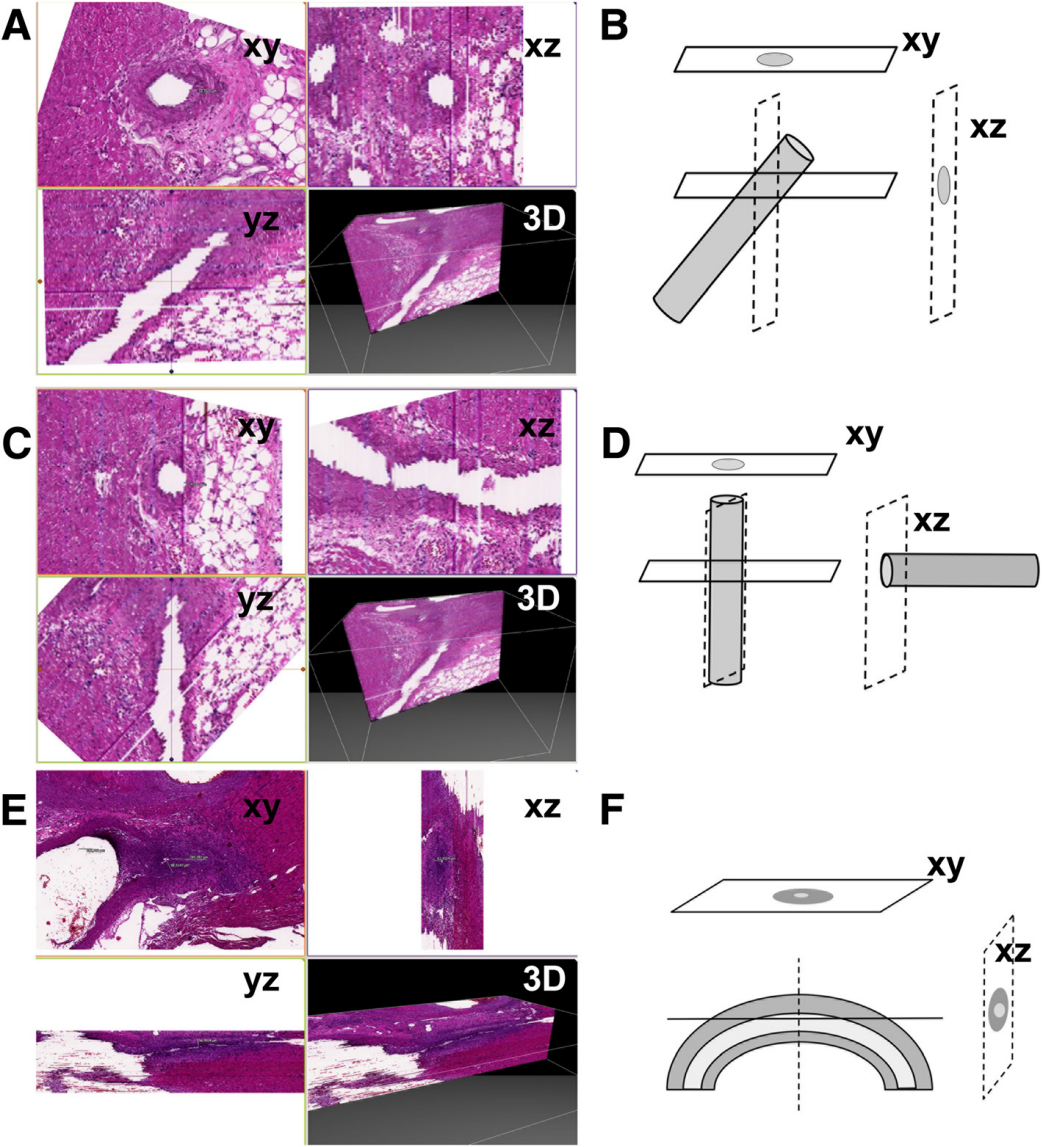
**3D reconstruction and virtual sectioning of coronaries. A** and **B**. 3D reconstruction (xy/xz/yz planes and 3D) and graphical representation of an obliquely cut vessel with minimal CAV. The xy plane (upper left) is digital image of the tissue as it appears after initial sectioning. **C**. After initial 3D reconstruction, the virtual specimen was rotated and sectioned digitally into true cross sections of the coronary artery (Upon rotation the image of the virtual planes are changing, the 3D object does not). **D**. Graphical representation of the virtual rotation and resampling. **E**. 3D reconstruction of a curved and obliquely cut coronary artery with severe CAV. In this situation, conventional histology is unlikely to obtain precise measurements of the NI. **F**. Graphical representation of a section plane with a falsely elevated NI, and a rotated virtual section plane with an accurate NI.

3D reconstruction of mouse hearts was performed using serial sectioning and digital slides. The μCore software enabled us to perform virtual sectioning of the 3D dataset and upon localizing areas of interest, technically virtual but practically true cross sections of the coronary arteries were obtained. Figure 2A-D. show an example of an obliquely cut vessel and how it was corrected using our method to visualize true cross sections. Figure 2E and F give an example of a curved and obliquely cut vessel. In this circumstance, no conventional methods are capable of performing precise measurements.

After detecting areas of interest and creating correct virtual cross sections, the NI of the coronary arteries, spanning 0.4 mm (Sample A) and 0.8 mm (Sample B) length of artery were calculated on altogether four times twenty-eight non-corrected and corrected images (two samples, two different method, 28 different levels). The results obtained from the corrected images were statistically different from those obtained from the non-corrected images [t(55) = −5.343, p = 2.0e-06]. Both conventional measurement method and reconstruction and virtual sectioning demonstrated greater NI in proximal segments of the coronary arteries relative to distal segments. Conventional measurements of both samples had neointimal indices of 86.31 ± 12.06 and 72.26 ± 10.00 for the proximal and distal parts, respectively (p = 1.7e-05). On the contrary, measurements on the reoriented sections demonstrate the NI decreasing from 88.71 ± 10.28 proximally to 88.51 ± 10.03 distally (p = 0.940). When considering both methods we have seen a significant difference between the measurements of the distal segment (conventional 70.62 ± 10.35 vs. reconstructed 97.80 ± 1.39) of the second sample (p < 0.001) (Table 1). In Sample-B the examined coronary portion was cut obliquely (Figure 2E-F) and as we hypothesized applying the conventional method resulted in greater variability of the NI, while the reconstruction gave accurate results.

**Table 1:** **Neointimal Indexes calculated with conventional methods and after 3-dimensional reconstruction**

	**Full length**			**Proximal part**			**Distal part**		
	**Conventional method**	**After reconst-ruction**	**p**	**Conventional method**	**After reconst-ruction**	**p**	**Conventional method**	**After reconst-ruction**	**p**
Sample A	75.24 ± 7.23	78.94 ± 3.36	0,421	76.31 ± 2.44	78.66 ± 1.37	0,975	73.68 ± 9.82	79.22 ± 4.63	0,265
Sample B	83.33 ± 16.18	98.28 ± 1.10	0,001	96.31 ± 9.00	98.77 ± 0.32	0,968	70.62 ± 10.35	97.80 ± 1.39	0,001
Sample A + B	77.09 ± 5.89	90.81 ± 13.63	0,001	86.31 ± 12.06	88.71 ± 10.28	0,833	72.26 ± 10.00	88.51 ± 10.03	0,001

3-D reconstruction also enables improved visualization of the anatomy and structural relationships of the coronary vasculature relative to 2-D sections. We distinguished two main coronary arteries originating from the mouse aorta, one of which divided into 2 branches shortly distal to the ostia. This is analogous to the anatomy of the human coronary arteries, namely the right coronary artery and the left main coronary artery, the latter of which gives rise to the left anterior descending artery and the circumflex artery. In affected animals, the aortic root was involved by mild-to-moderate non-occlusive CAV, with intimal proliferation increasing markedly in coronary ostia and the proximal coronary artery. We observe that CAV is most severe between the ostia and the major branch points, while lumens of the distal coronaries, and diagonal and marginal branches remained almost entirely patent. More samples are required to analyze whether this finding has relevance in the development and progression of CAV in the murine heart transplantation model.

### Neointimal volume index

**Figure 3:**
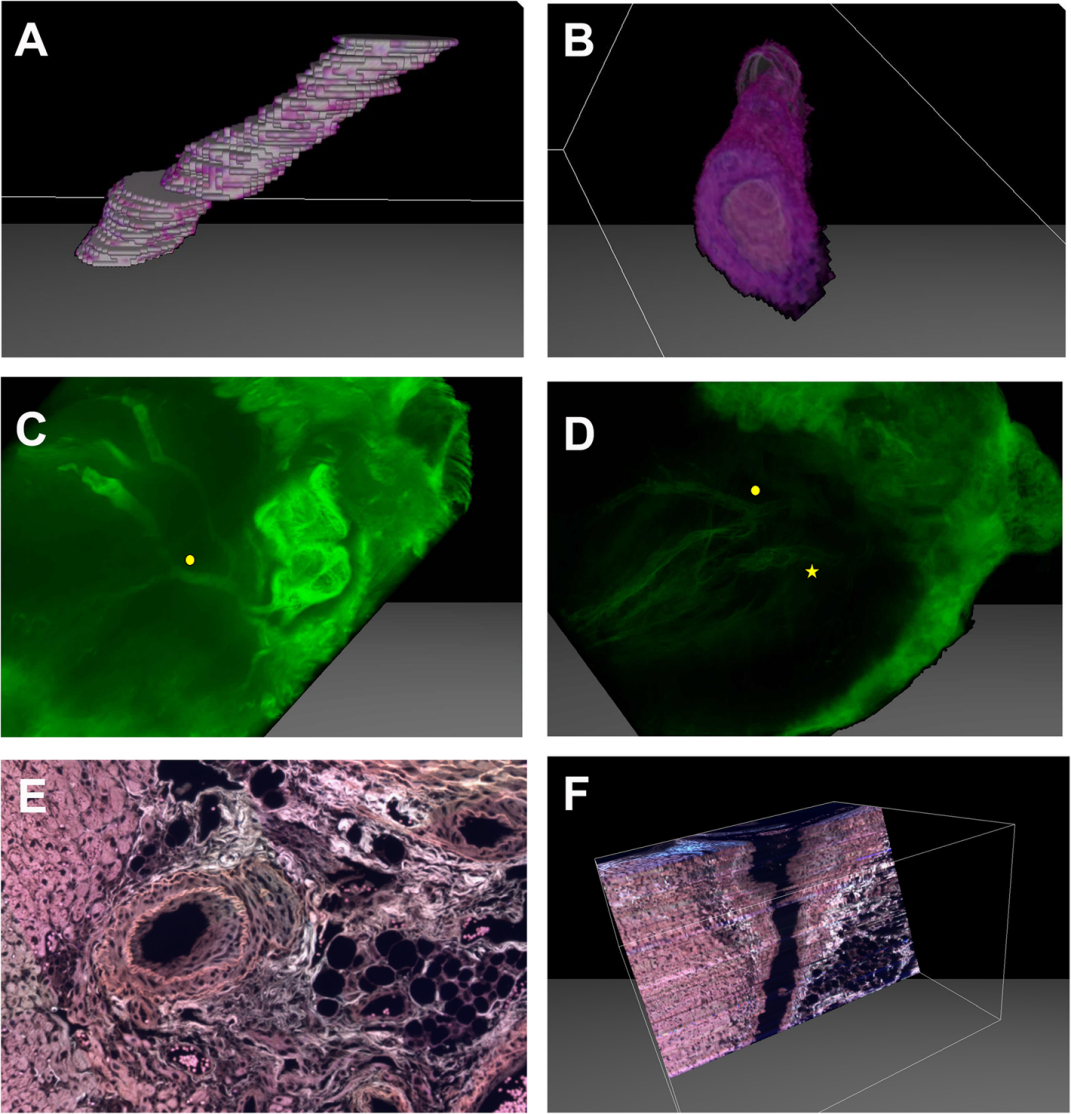
**Neointimal volume index, virtual coronary angiography.** Extracted lumen **(A)** and neointima **(B)** of a distal coronary portion from sample A. **C/D**: Through image modulation, color filtration and digital subtraction, the vasculature is highlighted and the background tissue suppressed, generating a virtual coronary angiography. (yellow circle: branching coronary – LAD, yellow star: non branching coronary – RCA.) **E**. Fluorescent scanning of an HE stained slide. The internal and external elastic lamina of the coronary artery are bright in this false color image, as are structures in the cardiac parenchyma with high collagen content. **F**. 3D reconstruction of the vasculature from HE stained slides imaged using fluorescence scanning.

Volumes of the neointima and lumen can be calculated once 3D images of the samples are reconstructed to obtain a novel assessment of CAV. Based on the original NI, we describe a new parameter: the neointimal volume index (NVI). This value is defined as neointimal volume divided by neointimal volume plus luminal volume multiplied by 100. To quantify the luminal volume and the neointimal volume of a vessel, we employed a segmentation algorithm on the volume data using the μCore software. A segmentation algorithm applies labels to all voxels (volumetric elements as a pendant to pixels) in the volumetric image. A subset of voxels in the volumetric image are initially manually assigned to lumen, neointima, or background. Based on these assignments, the segmentation algorithm automatically computes labels to all remaining voxels by optimizing an objective function based on volume gradients and color intensities. For accurate volumetric analysis of the neointima, extensive manual assignment of background voxels was needed due to the relatively flat intensity gradients compared to surrounding voxels. Figure 3 shows the extracted lumen (A) and neointima (B) of a distal coronary portion of Sample A. The NVI for the highlighted coronary portion is 59.705. (Vneointima = 153.952 voxels;Vneointima + lumen = 257.856 voxels)

.

### Virtual coronary angiography

Using multispectral and ultraviolet imaging, bandpass filtering, and digital subtraction techniques, 3-dimensional images of the complete coronary vasculature could also be obtained from serially sectioned H&E stained slides without the use of segmentation algorithms similar to a coronarography image (Figure 3C and D). A combination of DAPI, FITC, and Rhodamine filters were used to scan the H&E stained cardiac sections. Exposure times, excitation and emission wavelengths’ were as follows: 500 ms, 365 nm, 445 nm; 100 ms, 470 nm, 525 nm; 550 ms, 550 nm, 605 nm, respectively. As expected, fluorescent scanning resulted in strong autofluorescence of the collagenous structures. In particular, the elastic lamina could easily be discriminated from the surrounding structures (Figure 3E). Reconstructing 3D images of the vasculature by utilizing these digital fluorescent slides enables us to accurately assess neointimal thickness of the coronary arteries using true perpendicular cross-sections (Figure 3F).

The techniques of 3D image reconstruction are newly introduced modalities in the fields of medical imaging and provide increased diagnostic accuracy compared with conventional 2D imaging methods, especially in the positional relationship between the lesions and surrounding structures. The field of radiology has pioneered the use of 3D reconstruction from computed tomography and magnetic resonance images, offering intuitive and informative visualizations for diagnosing disease, surgical planning, radiation oncology treatment and other clinical applications. Pathology is another field in which 3D reconstruction offers the potential to serve as a useful scientific tool, revolutionize diagnosis, and improve clinical management. Recent technical advances in whole slide imaging (WSI) in pathology offer high throughput, high magnification and high resolution [14]. These technical breakthroughs permit the generation of high quality 3D reconstructions able to display the physical relationships between microscopic structures. Furthermore, immunohistochemistry and other stains can be applied to the sections before reconstruction and the 3D relationships of structures and cells can be revealed.

Currently, the challenges limiting the further adoption of 3D imaging of serial sections are physical artifacts from sectioning, digital artifacts resulting from the assembly and manipulation of 3D structures and their conversion to 2D image, and the time consuming and laborious process of cutting sections and digital reconstruction. These limitations require the researchers’ careful consideration on estimating costs and benefits when choosing 3D imaging of serial sections as a method to investigate scientific questions. Although other 3D imaging techniques such as optical coherence tomography (OCT), microcomputed tomography (microCT) or multi-photon fluorescence imaging are more rapid and preserve the original tissue for further analysis. However, each alternative has its own limitations, such as unsuitability to large samples (OCT), lower resolution (microCT) or detection limited to structures with defined excitation and emission characteristics (multi-photon imaging). The use of 3D reconstruction from serial sections is therefore a labor intensive but versatile tool that is most advantageous when analyzing larger portions of tissue for fine structural relationships between heterogeneous tissue types.

Tissue artifacts, including distortions, folds, fissures or gaps, are a normal consequence of sectioning and mounting samples on slides but present enormous challenges to accurate z-stack alignment, as reviewed by Braumann et al. [15]. Here, we attempted to limit artifacts by using automation to serially section the tissue. This resulted in tissue uniformly rotated and positioned on the slide, reduced tearing and fissuring artifacts, and maintained uniform stretching artifacts between tissue sections.

There is significant heterogeneity in the available approaches for capturing 2D images and reassembling them into virtual 3D structures. Many different hardware and software tools are commercially available for use in 3D reconstruction of serial sections of histological samples. A simple, low-cost approach exploits off-the-shelf hardware such as cameras attached to microscope heads to obtain photomicrographs of regions of interest, and freeware or low-cost software packages for image registration, alignment and stacking. Published reports using this technique have been able to obtain virtual reconstructions with an approximately 8 micrometer/pixel resolution, superior to the current generation of microCT devices [15].

The development of digital whole slide imaging uses automation to increase throughput and obtain images of higher resolution and uniform quality. The very first publication about WSI, besides describing the technique itself, already applied this new technique to reconstruct gastric malignancies in 3D images. This work was published in 1999, years before other groups reintroduced WSI as a tool for 3D reconstruction [16]. In the recent years, with the improvement of WSI [17] and with the design of automated serial sectioning devices [18], more papers are being published in this field. 3D reconstruction has been employed in the classification of lung adenocarcinomas, diagnosis of colorectal biopsies and metastasis of breast cancer to lymph nodes [19-21]. Roberts et al. from the University of Leeds reported a method using automatic image registration algorithms for 3D reconstructions that has been successfully applied to 8 different tissue types [22].

## Conclusions

In the current study, we demonstrate the feasibility of applying the WSI-3D technique to the reconstruction of the coronary vasculature in mouse hearts and demonstrate the applicability of this method to the research of chronic allograft rejection and the development of CAV. Although time consuming, this technique may actually increase efficiency, as the coronaries would be identified on all research specimens, and no specimens would be lost due to cutting through the tissue during blind sectioning. This technique would also ensure sampling of the proximal coronaries, the portions most likely to be affected by the development of CAV, thus reducing the false negative rate.

We show that virtual image rotation can obtain a true vascular cross-section, reducing artifacts encountered during conventional 2-dimensional image assessments to measure NI, and therefore increasing scientific accuracy. By integrating the neointimal index along a length of vessel to obtain a “neointimal volume index” (NVI), 3D reconstruction further improves accuracy over conventional NI by eliminating sampling bias.

Finally, although autofluorescence is typically an undesired imaging artifact in tissues with high collagen content, we convert it into a useful marker capable of highlighting vascular structures on H&E and even unstained slides. This method is capable of localizing areas of interests for automated high resolution reconstruction, and can be used to identify which unstained slides contained sections of the proximal coronaries for further analysis with immunohistochemical stains.

We believe 3D reconstruction of 2D histological slides will provide new insights into pathological mechanisms in which structural abnormalities play a role in the development of a disease. The techniques we describe are applicable to the analysis of arteries, veins, bronchioles and similar sized structures in a variety of tissue types and disease model systems.

## Abbreviations

CAV: Chronic allograft vasculopathy
microCT: Microcomputed tomography
NI: Neointimal index
NVI: Neointimal volume index
OCT: Optical coherence tomography
WSI: Whole slide imaging
3D: Three-dimensional

## References

[1] Russell PS, Chase CM, Winn HJ, Colvin RB (1994). Coronary atherosclerosis in transplanted mouse hearts.2. importance of humoral immunity. J Immunol.

[2] Russell PS, Chase CM, Colvin RB (1997). Alloantibody- and T cell-mediated immunity in the pathogenesis of transplant arteriosclerosis - Lack of progression to sclerotic lesions in B cell-deficient mice. Transplantation.

[3] Uehara S, Chase CM, Kitchens WH, Rose HS, Colvin RB, Russell PS (2005). NK cells can trigger allograft vasculopathy: the role of hybrid resistance in solid organ allografts. J Immunol.

[4] Uehara S, Chase CM, Colvin RB, Madsen JC, Russell PS (2006). T-Cell depletion eliminates the development of cardiac allograft vasculopathy in mice rendered tolerant by the induction of mixed chimerism. Transplant Proc.

[5] Uehara S, Chase CM, Cornell LD, Madsen JC, Russell PS, Colvin RB (2007). Chronic cardiac transplant arteriopathy in mice: relationship of alloantibody, C4d deposition and neointimal fibrosis. Am J Transplant.

[6] Hirohashi T, Uehara S, Chase CM, DellaPelle P, Madsen JC, Russell PS (2010). Complement independent antibody-mediated endarteritis and transplant arteriopathy in mice. Am J Transplant.

[7] Hirohashi T, Chase CM, Della Pelle P, Sebastian D, Alessandrini A, Madsen JC (2012). A novel pathway of chronic allograft rejection mediated by NK cells and alloantibody. Am J Transplant.

[8] Armstrong AT, Strauch AR, Starling RC, Sedmak DD, Orosz CG (1997). Morphometric analysis of neointimal formation in murine cardiac allografts. Transplantation.

[9] Armstrong AT, Strauch AR, Starling RC, Sedmak DD, Orosz CG (1997). Morphometric analysis of neointimal formation in murine cardiac allografts .2. Rate and location of lesion development. Transplantation.

[10] Russell PS, Chase CM, Sykes M, Ito H, Shaffer J, Colvin RB (2001). Tolerance, mixed chimerism, and chronic transplant arteriopathy. J Immunol.

[11] Corry RJ, Winn HJ, Russell PS (1973). Primarily vascularized allografts of hearts in mice. The role of H-2D, H-2 K, and non-H-2 antigens in rejection. Transplantation.

[12] Eichwald EJ, Silmser CR (1955). Skin. Transplant Bull.

[13] Russell PS, Chase CM, Madsen JC, Hirohashi T, Cornell LD, Sproule TJ (2011). Coronary artery disease from isolated non-H2-determined incompatibilities in transplanted mouse hearts. Transplantation.

[14] Weinstein RS, Graham AR, Lian F, Braunhut BL, Barker GR, Krupinski EA (2012). Reconciliation of diverse telepathology system designs. Historic issues and implications for emerging markets and new applications. APMIS.

[15] Braumann UD, Kuska JP, Einenkel J, Horn LC, Loffler M, Hockel M (2005). Three-dimensional reconstruction and quantification of cervical carcinoma invasion fronts from histological serial sections. IEEE Trans Med Imag.

[16] Molnar B, Papik K, Tagscherer A, Tulassay Z (1999). Three-dimensional reconstruction and analysis of gastric malignancies by electronic slides of consecutive sections and virtual microscopy. Proceedings of SPIE.

[17] Al-Janabi S, Huisman A, Vink A, Leguit RJ, Offerhaus GJA, ten Kate FJW (2012). Whole slide images for primary diagnostics in dermatopathology: a feasibility study. J Clin Pathol.

[18] Onozato ML, Hammond S, Merren M, Yagi Y (2011). Evaluation of a completely automated tissue-sectioning machine for paraffin blocks. J Clin Pathol.

[19] Onozato ML, Klepeis VE, Yagi Y, Mino-Kenudson M (2012). A role of three-dimensional (3D)-reconstruction in the classification of lung adenocarcinoma. Anal Cell Pathol.

[20] Wu MLC, Varga VS, Kamaras V, Ficsor L, Tagscherer A, Tulassay Z (2005). Three-dimensional virtual microscopy of colorectal biopsies. Arch Pathol Lab Med.

[21] Paish EC, Green AR, Rakha EA, Macmillan RD, Maddison JR, Ellis IO (2009). Three-dimensional reconstruction of sentinel lymph nodes with metastatic breast cancer indicates three distinct patterns of tumour growth. J Clin Pathol.

[22] Roberts N, Magee D, Song Y, Brabazon K, Shires M, Crellin D (2012). Toward routine use of 3D histopathology as a research tool. Am J Pathol.

